# Nasal citology: a new diagnostic approach in rhinology

**DOI:** 10.1186/2045-7022-5-S4-P5

**Published:** 2015-06-26

**Authors:** Alberto Macchi, Paola Terranova, Giulia Montrasio, Matteo Gelardi, Eleonora Sica

**Affiliations:** 1 ORL Clinic University of Insubria, Varese, Italy; 2 University of Insubria Varese, ORL Clinic, Varese, Italy; 3 University of Bari, ORL Clinic, Bari, Italy

## 

Nasal cytology is a diagnostic method in the field rinology used to detect changes into the endonasal epithelium exposed to physical and/or chemical irritation, acute or chronic inflammation due to different angents (viral, bacterial, fungal or parasitic). Nasal cytology was born in 1889 thanks to H. Gollash who found many eosinophilic cells into the nasal secretions of a patient affected by asthma, and thought of them as important elements in the pathogenesis of this disease. The main evolution of nasal cytology occurred in 1927, thanks to the report done by C. Eyermann, who noted the presence of eosinophilic cells into the nasal secretions of allergic patients. Since then great importance is given to the recognition of specific cell types into the pathogenesis of different nasal diseases. Nasal cytology is nowadays more frequently used in the study of allergic, vasomotor, inflammatory and infectious rhinitis. It is a simple, non-invasive and repeatable examination which is useful in the follow-up and monitoring of the real effectiveness of medical and surgical treatments. Patients affected by allergic rhinitis (AR) develop an endonasal immediate response, so-called “early phase”, followed by a “late phase” response to the allergenic agent. From the microscopic point of view, both these responses are always characterized by an infiltration of the mucosa by immunoflogistic cells (eosinophils, mast cells, neutrophils and lymphocytes) that, following the release of several chemical mediators, are the main cause of the symptoms that characterize the IgE-mediated disease. In AR the main cells type released during the immunological response will change depending on whether the patient is examined during or outside the pollinic season. In the first condition, the patient will present all the clinical signs and his nasal cytology examination will be characterized by neutrophils, lymphocytes, eosinophils and mast cells largely degranulated. Nasal cytology allows us to differentiate between various forms of rhinitis which are resumed as: non-allergic rhinitis with neutrophil (Narne); non-allergic rhinitis with eosinophilia (NARES); non-allergic rhinitis with mast cells (NARMA); non-allergic eosino.

